# Delayed migration of a peripherally inserted central venous catheter to the azygos vein with subsequent perforation

**DOI:** 10.1259/bjrcr.20150315

**Published:** 2016-02-10

**Authors:** Rukshan Fernando, Yu Jin Lee, Nasir Khan, Farhat Kazmi

**Affiliations:** ^1^ Department of Radiology, Chelsea and Westminster Hospital, London, UK; ^2^ Department of Radiology, Royal Brompton Hospital, London, UK; ^3^ Department of Radiology, Royal Marsden Hospital, London, UK

## Abstract

Peripherally inserted central venous catheters (PICCs) are often used for infusion of chemotherapeutic agents, long-term antibiotics or total parenteral nutrition (TPN). We present a case of delayed migration of a PICC inserted for TPN from the superior vena cava into the azygos vein that was not initially recognized on chest radiographs or CT scan. This subsequently led to azygos perforation and extravasation of the TPN solution into the mediastinal, pleural and pericardial spaces. Several anatomical and procedural factors predispose to PICC migration. In this patient, the risk of PICC migration was increased by left-sided insertion and variant azygos anatomy. If a curve in the distal tip of a PICC is seen on a frontal chest radiograph, azygos malposition should be suspected and confirmed with a lateral radiograph, CT scan or catheter injection. This is because azygos malposition increases the risk of venous perforation and needs to be corrected.

## Case presentation

A 21-year-old female with primary ciliary dyskinesia and situs ambiguus was admitted electively for a diagnostic laparoscopy because of a history of recurrent subacute bowel obstructions. A pre-operative CT scan of her chest and abdomen showed that the heart was normally sited but the abdominal viscera were inverted. There was also polysplenia and absence of the suprarenal inferior vena cava (IVC). The infrarenal IVC was instead continuous with the azygos vein, with the resultant increased flow causing azygos dilatation. A further anatomical variant that was present was the azygos opening into the right lateral wall of the superior vena cava (SVC) instead of its usual drainage into the posterior wall.

Intraoperatively, extensive jejunal adhesions were found, which required conversion from a laparoscopic to an open procedure. Adhesiolysis and appendicectomy were performed. In the early post-operative period, the patient had a prolonged ileus with intractable vomiting and high nasogastric tube output. As she was unable to tolerate oral nutrition, it was decided to start her on total parenteral nutrition (TPN), for which a peripherally inserted central venous catheter (PICC) insertion was requested.

**Figure 1.**
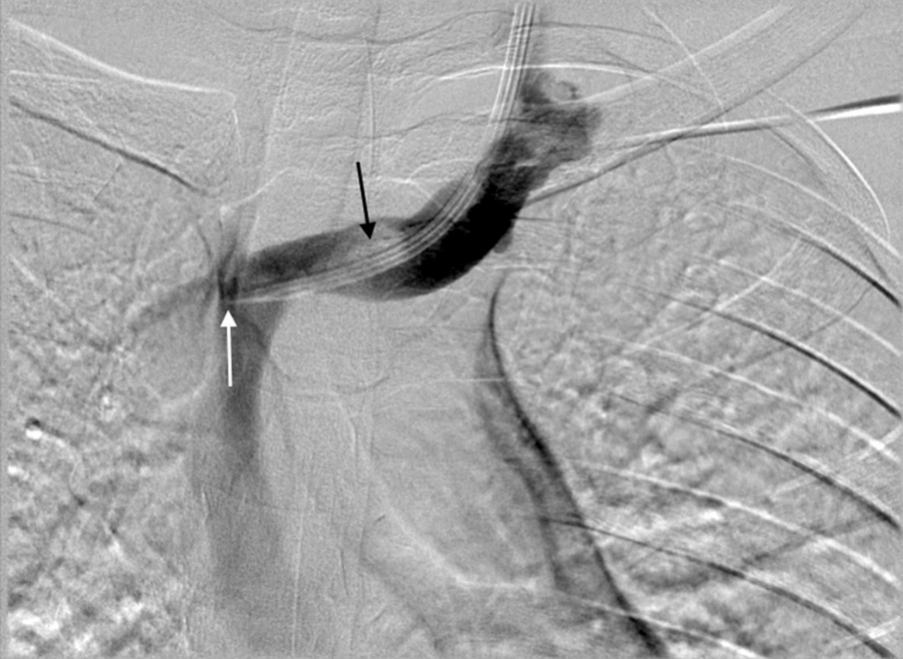
Digital subtraction angiogram performed during peripherally inserted central venous catheter insertion demonstrates a filling defect in the left brachiocephalic vein (black arrow), representing a thrombus. A left internal jugular vein central line is *in situ* (white arrow).

**Figure 2.**
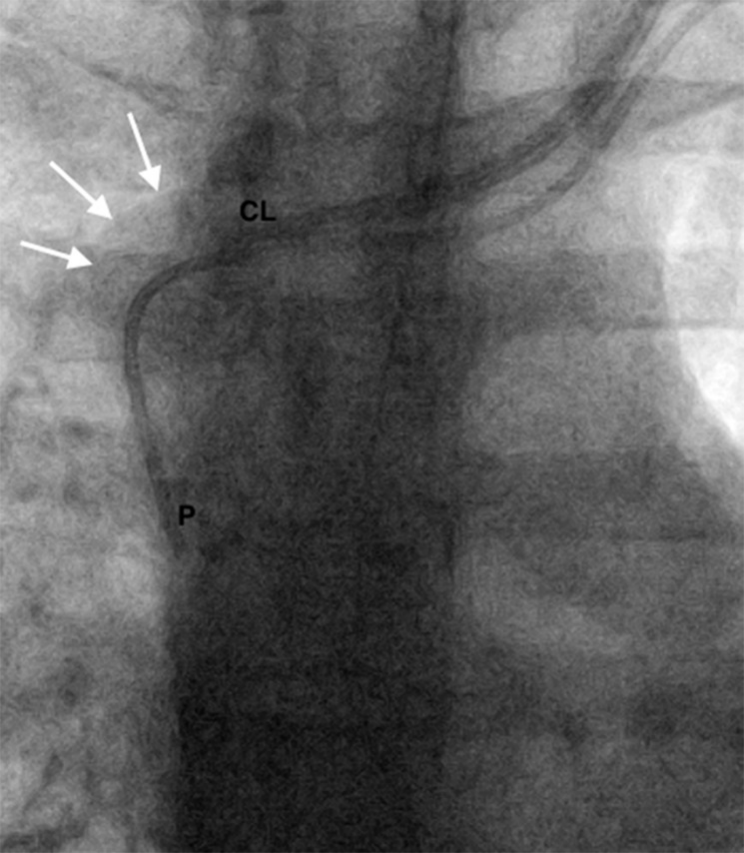
Fluoroscopic spot image obtained during PICC insertion shows the tip of the PICC (P) in the distal superior vena cava. The white arrows denote the dilated azygos vein. CL, left internal jugular vein central line; PICC, peripherally inserted central venous catheter.

The PICC was inserted under fluoroscopic guidance using a modified Seldinger technique by a radiology registrar and consultant interventional radiologist. The left arm was abducted to 90° during insertion and venous access was obtained via the left basilic vein. During the procedure, there was difficulty advancing the nitinol guidewire into the SVC. A digital subtraction angiogram was performed that demonstrated thrombus in the left brachiocephalic vein (Figure 1). The nitinol guidewire was exchanged for a 0.018” Terumo Radifocus^®^ guidewire (Terumo, Tokyo, Japan) that passed through the thrombus without difficulty into the SVC. A 5 French Cook Turbo-Ject^®^ (Cook Medical, Bloomington, IN) double-lumen polyurethane PICC was then inserted over the guidewire with its tip sited in the distal SVC (Figure 2). Blood was readily aspirated from the PICC and the line was flushed and secured. To prevent excessive catheter length outside the patient, the distal portion of the PICC was cut before insertion.

**Figure 3.**
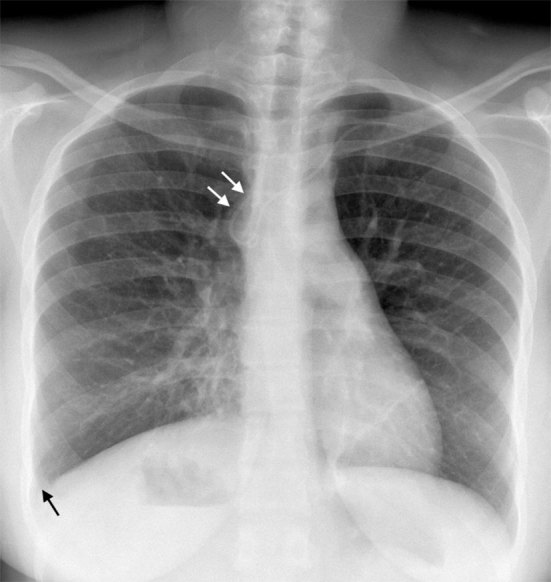
Frontal chest radiograph 9 days after peripherally inserted central venous catheter insertion shows a small right-sided pleural effusion (black arrow) and a curve in the distal catheter tip (white arrows). The heart is normally oriented but the gastric bubble is under the right hemidiaphragm, consistent with situs ambiguus.

TPN was commenced the same day at a rate of 1.5–2.5 l day^–1^, and a left internal jugular vein (IJV) line that had been *in situ* was removed. Over the following week, the patient’s ileus gradually resolved. On the ninth day following PICC insertion, the patient experienced dyspnoea and pleuritic chest pain. A chest radiograph was performed (Figure 3) that showed a small right-sided pleural effusion. A curve at the distal end of the PICC was also noted, which was reported as coiling of the PICC within the SVC. TPN infusion was continued because the PICC could still be aspirated and flushed.

**Figure 4.**
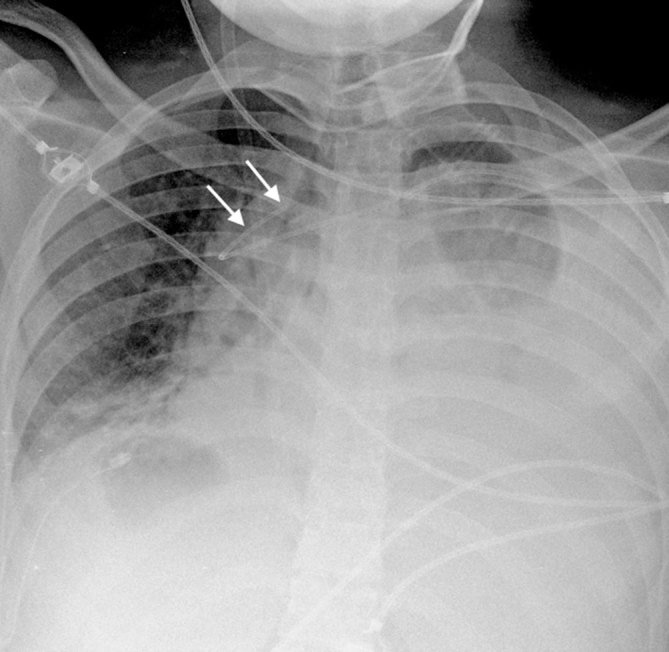
Frontal chest radiograph 10 days after PICC insertion shows large bilateral pleural effusions with atelectasis. The PICC tip remains curved (white arrows). PICC. peripherally inserted central venous catheter.

Over the next 24 h, the patient deteriorated further and became tachycardic, tachypnoeic and hypoxic. A repeat chest radiograph was obtained that showed large bilateral pleural effusions (Figure 4). The PICC remained in the same position as the previous day. A CT pulmonary angiogram (CTPA) was requested to exclude a pulmonary embolus (PE) and look for a cause for the rapidly increasing pleural effusions.

**Figure 5.**
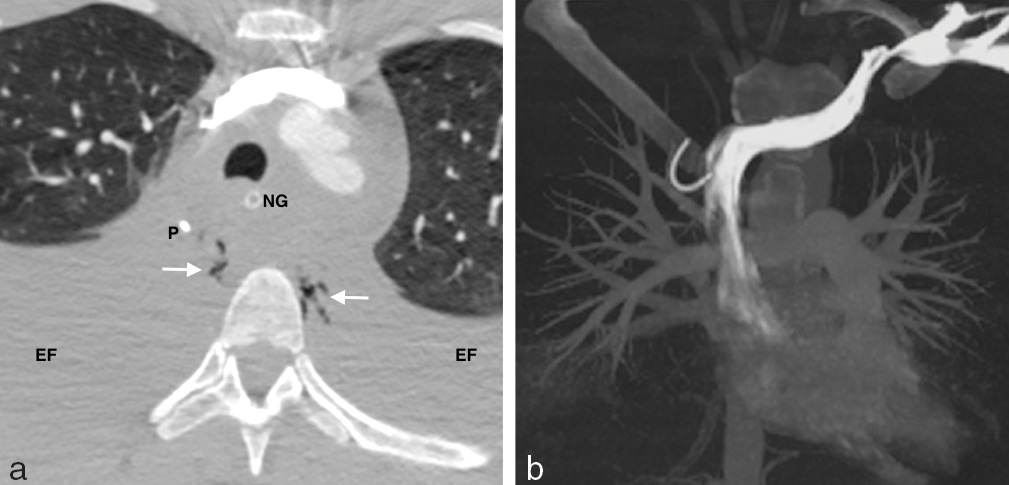
(a) Axial slice from the CTPA shows large bilateral pleural effusions (EF) and pneumomediastinum (white arrows). A nasogastric tube (NG) is sited within the oesophagus, which is displaced anteriorly together with the trachea by a large mediastinal effusion. The PICC tip (P) is adjacent to locules of mediastinal gas. The azygos is not clearly seen on this image, as it is isodense to the surrounding effusion. (b) Coronal three-dimensional maximum intensity projection reconstruction of the CTPA shows the PICC displaced outside the superior vena cava. CTPA, CT pulmonary angiogram; PICC, peripherally inserted central venous catheter.

The CTPA was negative for a PE but did demonstrate large bilateral pleural effusions, a moderate-sized pericardial effusion, a mediastinal effusion and pneumomediastinum (Figure 5a,b). The PICC was again reported as being coiled within the SVC and repositioning was advised.

An echocardiogram performed to assess the pericardial effusion showed no evidence of tamponade. In view of the large bilateral pleural effusions, a pleural aspiration was performed. Milky white fluid was obtained, similar in appearance to TPN solution. There was no blood in the aspirate. Biochemical analysis confirmed that the aspirated fluid was TPN solution and not chyle. This raised the suspicion of vascular perforation by the PICC.

**Figure 6.**
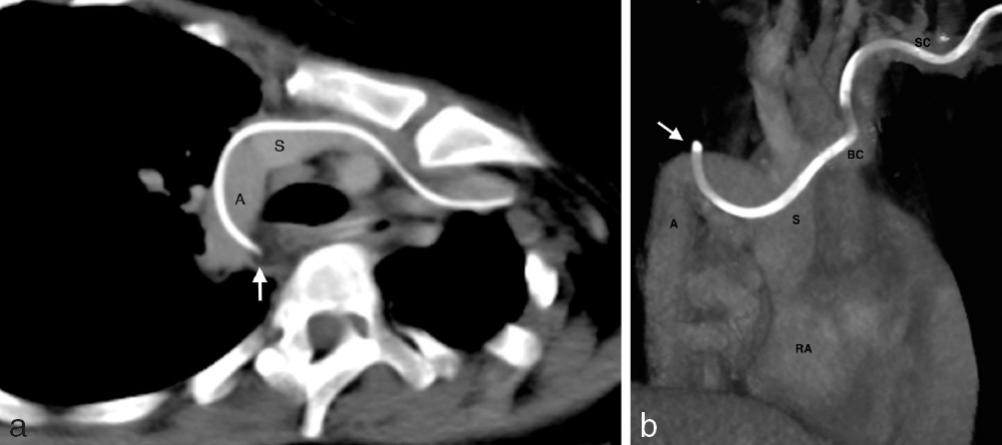
(a) Oblique axial slice from the CT scan obtained after drainage of the effusions shows that the PICC tip (white arrow) has perforated the wall of the azygos (A). The azygos is dilated and has a similar diameter to the SVC (S). (b) Coronal three-dimensional reconstruction also demonstrating perforation of the azygos (A) by the PICC (white arrow). There is variant drainage of the azygos into the right lateral wall of the SVC (S). This creates a favourable angle for migration of a left-sided PICC into the azygos. BC, left brachiocephalic vein; PICC, peripherally inserted central venous catheter; RA, right atrium; SC. left subclavian vein; SVC, superior vena cava.

The patient was transferred to a cardiothoracic centre and bilateral intercostal chest drains were inserted. A total of 2 l of TPN solution was removed. A repeat CT scan showed that the PICC was not actually coiled within the SVC, but had migrated to the azygos and caused a perforation (Figure 6a,b). The cardiothoracic surgeons decided to remove the PICC with close observation afterwards for any signs of deterioration. The patient remained clinically stable and made a good recovery. She was discharged home a week after removal of the PICC.

## Discussion

The use of PICCs in both inpatient and outpatient settings has increased in recent years. PICCs have several advantages over central venous catheters (CVCs) including decreased risk of arterial injury and pneumothorax.^1^ However, as demonstrated in this case, complications such as catheter migration and perforation can occur and need to be recognized early.

Catheter migration to the azygos is rare, occurring in approximately 1% of CVC insertions, and can occur as either an early or late complication.^2^ The average time for catheter migration reported in a case review by Haygood et al was 43 days.^3^ Venous perforation and cardiac tamponade following PICC insertion are also relatively rare, with only a few reported cases in the literature.^4,5^

In this discussion, the factors that increase the risk of PICC migration and perforation will be described. The key imaging findings to note in azygos malpositioning are also highlighted.

### Risk factors for peripherally inserted venous catheter migration

The risk factors for PICC migration can be subdivided into anatomical, procedural and post-procedural factors.

#### Anatomical

The left brachiocephalic vein passes in an oblique anteroposterior direction as it joins the right brachiocephalic vein to form the SVC. The azygos arches over the right main bronchus posteroanteriorly before opening into the posterior wall of the SVC. There is a relatively shallow angle between the openings of the left brachiocephalic and azygos veins into the SVC. Hence, a PICC inserted from the left, as was the case here, is more likely to end up in the azygos than one inserted from the right.^3^ In this patient, the azygos opened into the right lateral wall of the SVC, creating an even more favourable angle for left-sided PICC migration (Figure 6b).

#### Procedural

Use of image guidance and depth of catheter placement are the main procedural factors that influence the risk of PICC migration. PICC insertions are performed either with fluoroscopy or at the bedside with a post-procedural chest radiograph to check the position of the catheter tip. Fluoroscopic-guided insertion offers the advantage of real-time visualization of the guidewire and catheter, allowing catheter malpositions to be immediately corrected. Bedside insertions have been shown to have a 10% incidence of malposition on the initial post-procedural radiograph.^1^ The usual location for placing the catheter tip is the distal third of the SVC, near the cavo-atrial junction, because this theoretically reduces the risk of migration into the azygos or the right atrium. However, a case review performed by Haygood et al did not show a correlation between the depth of catheter placement and the risk of subsequent migration.^3^

#### Post-procedural

One of the risk factors for post-procedural PICC migration is movement of the catheter with changes in arm or body position. PICCs inserted in an abducted arm have been shown to migrate caudally by an average of 2 cm in adduction.^6^ Cranial migration can be caused by changing from a supine to standing position. Another possible post-procedural cause for catheter migration in this patient was removal of the left IJV CVC after the PICC was inserted. The CVC could have snared the PICC during removal, causing displacement into the azygos.

### Risk factors for venous perforation

There is an increased risk of perforation when the PICC is sited within the azygos because of the smaller diameter of the azygos compared with the SVC.^2^ However, this factor was unlikely to have been significant in this patient because her azygos was dilated. A more likely cause of perforation was the orientation of the PICC to the vessel wall. The PICC should lie parallel to the long axis of the vein.^7^ If it lies obliquely or perpendicularly, as is usually the case with azygos malpositions, then movement of the PICC with changes in body position may cause the tip to repeatedly abut the vessel wall, eventually causing a small perforation.^4^

The catheter material and stiffness may also influence the risk of perforation. Softer materials such as silicone and polyurethane have been shown in laboratory models to have a lower risk of perforation than stiffer materials such as polyethylene.^8^ Another factor increasing the risk of perforation in this case was the cutting of the distal catheter before insertion, resulting in a sharper tip that could erode the vessel wall. Because of this, some authors advise against cutting a PICC to shorten it.^4^

### Recognizing azygos malpositions

Haygood et al^9^ analyzed the appearances of 30 posteroanterior chest radiographs that demonstrated azygos malpositioning of a CVC. In all the 30 cases, there was a curve at the distal end of the catheter (Figure 3). The direction of catheter tip deviation was variable. Rightward deviation was the most common, although the catheter could also be seen deviating to the left or pointing directly backwards.

A distally curved catheter does not always imply azygos malposition and could merely be coiled back on itself in the SVC, as was initially interpreted in this case. To differentiate between a coiled catheter and azygos malposition, a lateral chest radiograph should be obtained. A malpositioned catheter will be seen lying anteroposteriorly within the azygos arch. A CT scan can also be obtained to confirm the position of the catheter tip. If there is further doubt about the location of the catheter, contrast can be injected through the catheter while screening with fluoroscopy.

In this case, the distal curve in the catheter was noted on both the chest X-ray and the CTPA, but was not recognized as a possible azygos malposition. The possibility of venous perforation was also not raised in the CTPA report, despite the presence of large intrathoracic effusions. A possible reason for this oversight was that, on the CTPA, the azygos appeared isodense to the surrounding tissues because of fluid within the mediastinal and pleural spaces (Figure 5a). On the repeat CT scan performed following drainage of the effusions, the azygos perforation was more clearly seen (Figure 6a). Nevertheless, even on the CTPA, the catheter tip could be seen outside the SVC (Figure 5b) that should have raised a concern for malposition or perforation.

Once an azygos malposition has been recognized, the referring clinician needs to be informed and the communication should be documented. If there is concern that perforation may have occurred, such as in a haemodynamically unstable patient with new-onset pericardial or pleural effusions, then the advice should be to leave the catheter in place and consult a vascular surgeon or an interventional radiologist.^7^ This is because the catheter may be occluding the defect in the vessel wall and repositioning or removal can lead to massive haemorrhage.

## Learning points

1. PICCs inserted from the left are more likely to migrate to the azygos.
2. If a curve in the distal catheter is seen on a frontal radiograph, azygos malposition should be suspected. This can be confirmed on a lateral radiograph or CT scan.
3. It is important to recognize azygos malposition and inform the referring clinician because it is associated with an increased risk of venous perforation.
4. If perforation is suspected, the catheter should not be repositioned or removed. A vascular surgeon or an interventional radiologist should be consulted.
